# Sodium Butyrate Alleviates Heat Stress-Induced Oxidative Stress and Skeletal Muscle Homeostasis Disruption by Promoting Autophagy in Mice

**DOI:** 10.3390/nu17040696

**Published:** 2025-02-15

**Authors:** Jiayin Lu, Chaoyue Li, Tong Zhao, Fengyang Li, Zhichao Yao, Yajie Dong, Zeen Gong, Yi Yan, Xiaomao Luo, Haidong Wang

**Affiliations:** College of Veterinary Medicine, Shanxi Agricultural University, No.1 Mingxian South Road, Taigu 030801, China; l2473922146@163.com (C.L.); zhaotong20212402@163.com (T.Z.); lfy00917@163.com (F.L.); 15235484382@163.com (Z.Y.); dyj18335480794@163.com (Y.D.); 18404983660@163.com (Z.G.); yanyi@sxau.edu.cn (Y.Y.); xmluo@sxau.edu.cn (X.L.)

**Keywords:** heat stress, NaB, gut microorganism, skeletal muscle, autophagy, oxidative stress

## Abstract

Background: The gradual rise in global temperatures can affect skeletal muscle development and intestinal microorganisms. However, the influence of microbial metabolites on skeletal muscle homeostasis under heat stress (HS) remains unclear. Methods: C57BL/6J mice were exposed to normal temperature or 40 °C conditions for 3 d, 7 d, or 14 d. The HS 7 d mice also were treated with sodium butyrate (NaB, 200 mg/kg, gavage). Results: Strikingly, the body weight, antioxidative ability (MDA, T-SOD, and GSH-Px), and average cross-sectional area decreased, but the blood glucose and core temperature increased under HS. However, the NaB treatment reversed these effects. Meanwhile, HS also increased the levels of TNF-α and CORT. Additionally, HS led to a reduction in the villus height and an increase in the crypt depth of the intestine. Microbial 16S rRNA sequencing analysis revealed that HS caused gut microbiota dysbiosis. NaB increased the expression of HSP70 under HS, to maintain skeletal muscle homeostasis. HS stimulated the expression of Pax7, which indicates that skeletal muscle homeostasis was disrupted. Meanwhile, the expressions of MyoG and MyoD were decreased under HS. The immunofluorescence results also show that HS triggered a shift from slow muscle fibers (MYH7) to fast muscle fibers (MYH1). However, NaB recovered the expressions of these muscle-related factors. HS inhibited autophagy initiation (mTOR, Beclin1, Atg5, Atg7, and Atg12), the formation (LC3 II/LC3 I) of autophagosomes, and the binding (p62 and LAMP1) of lysosomes to autophagosomes, which were activated by NaB. C2C12 cells were treated with H_2_O_2_ to simulate skeletal muscle oxidative stress, and treated with NaB in advance. Oxidative stress disrupted the homeostasis of the C2C12 cells, characterized by an increase in Pax 7 and decreases in MyoG and MyoD, but these changes were reversed by the NaB treatment. Meanwhile, NaB was unable to maintain the stable expression of Pax7 when autophagy was inhibited. Conclusions: This suggests that NaB can regulate oxidative stress induced by HS through autophagy to maintain skeletal muscle homeostasis.

## 1. Introduction

In recent years, the frequency of extreme heat events (EHEs) has been increasing [1]. Intense high temperatures cause higher mortality rates, and EHEs will continue for a long time [1,2,3]. To a certain extent, high temperatures cause an enormous stress response in livestock, with harmful effects on animal meat product quality [4]. Heat stress (HS) occurs when the sensible temperature surpasses an animal’s thermoregulatory capacity, adversely affecting its health and growth [5]. Furthermore, heat stress affects organism development and metabolism [6]. As is widely known, the metabolic level is an important factor in maintaining muscle homeostasis and intestinal function. Recently, a study showed that humid heat affected emotion by disrupting gut microbiota and microbiota metabolism [7]. Additionally, heat stress can also cause decreases in the muscle antioxidant capacity, affecting the muscle protein metabolism of broilers [8]. Intestinal flora can influence the muscular immune response in Duchenne muscular dystrophy (DMD) [9]. Studies have shown that intestinal flora is an important target of astaxanthin in protecting against skeletal muscle atrophy [10]. Therefore, there is a strong correlation between gut microbiota and skeletal muscle homeostasis. However, the mechanism by which intestinal microbial metabolites affect skeletal muscle homeostasis remains unclear.

The development of skeletal muscle is important for maintaining posture and promoting metabolic balance. However, HS can cause increased proteolysis and influence the growth performance, and oxidative status, of skeletal muscle [8,11]. Thus, HS disrupts the homeostasis of skeletal muscle and destroys muscle function, which influences the body’s development and postural stability. When exposed to extreme heat, the levels of HSP70 and Pax7 protein in skeletal muscle cells increase significantly, affecting skeletal muscle homeostasis [12]. HS can also inhibit the proliferation and differentiation of C2C12 cells, triggering a mitochondria-related mechanism [13]. Mitochondria are key sites of redox reactions in the body. Thus, HS may induce oxidative stress in skeletal muscle. After 12 h of HS, the oxidative stress marker malondialdehyde (MDA) increased, while the catalase activity of the semitendinosus was reduced. Additionally, autophagy appeared to be inhibited [14]. Moreover, the phosphorylation of mTOR was increased, further inhibiting autophagy, which increased the levels of heat shock protein (HSP) 25, HSP40, HSP60, and HSP70 and inhibited the proliferation of skeletal muscle [15,16]. Therefore, heat stress may regulate skeletal muscle development through autophagy.

Dietary intervention to restore intestinal health has attracted more and more attention due to the significant effect of heat stress on intestinal nutrition metabolism [17]. For example, emerging evidence suggests that metabolite butyrate, a short-chain fatty acid (SCFA) produced by gut microbiota, can balance the synthesis and degradation of muscle proteins to alleviate muscle atrophy [18]. Butyrate can improve the growth performance of piglets [19] and facilitate autophagy by inhibiting the mammalian target of rapamycin (mTOR) signaling pathway in bovine skeletal muscle satellite cells (BSCs) [20], while acute HS promotes apoptosis and autophagy [21]. Butyrate also ameliorated muscle atrophy in diabetic nephropathy by activating the free fatty acid 2 (FFA2)-regulated phosphatidylinositol 3-kinase (PI3K)/Protein Kinase B (Akt)/mTOR signaling pathway [22]. However, the autophagy mechanism through which butyrate improves the development of skeletal muscle under HS is unclear.

The intestinal tract and skeletal muscle were the main objects of this study, and their morphological characteristics’ changes and flora composition under HS were investigated. The relationship between the intestinal tract and skeletal muscle was studied using the intestinal microbial metabolite NaB as a linker, and the mechanism of the regulation of intestinal microbial metabolites on skeletal muscle homeostasis was explored from the point of view of cellular autophagy.

## 2. Materials and Methods

### 2.1. Animal Model

**Figure 1:**
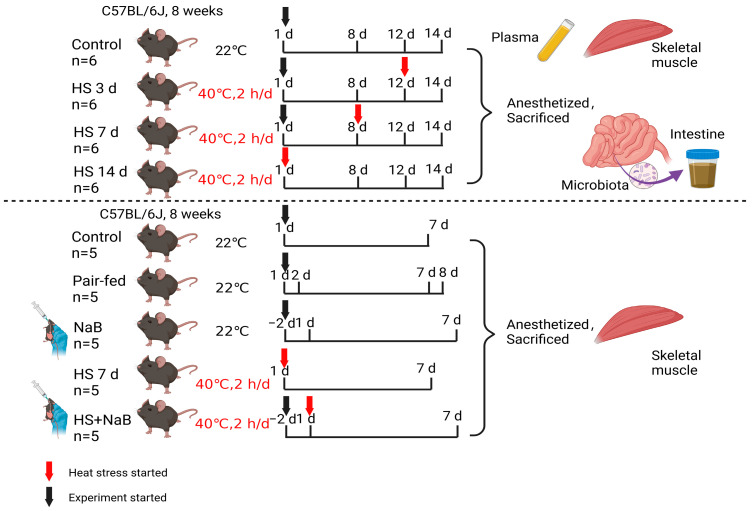
The design of animal experiments. The arrow represents the start time of the experiment, the red indicates the time when heat stress begins, and the black arrow represents the start time of the experiment for the non-stress group or the time when gavage begins.

In this work, the Animal Center of Shanxi Medical University (Taiyuan, Shanxi, China) provided 7-week-old C57BL/6J mice. The mice were maintained at a temperature of 21 ± 1 °C and relative humidity of 50 ± 10%. They were fed a standard diet (XTC01WC−001, Jiangsu Xietong Pharmaceutical Bio-engineering Co., Ltd., Nanjing, China) and kept on a regular 14 h:10 h (light/dark) cycle. To minimize potential confounders, feeding schedules were standardized to avoid circadian influences on metabolism. The individual mouse was considered the experimental unit within the studies. After a one-week adaptation period, no weak mice were observed. The 24 mice were randomly divided into four groups: control (*n* = 6), heat stress (HS) for 3 days (*n* = 6), HS 7 d (*n* = 6), and HS 14 d (*n* = 6). In the heat stress groups, the mice were moved to a chamber with a high temperature (40 °C) for 2 h every day. Then, 20 mice were randomly divided into three groups: control (*n* = 5), NaB (*n* = 5), HS 7 d (*n* = 5), and HS + NaB 7 d (*n* = 5; the NaB was administered intragastrically (200 mg/kg/d [23,24,25])) (Figure 1). Additionally, another random pair-fed (PF, *n* = 5) group was included to account for the effect of food on the mice in the treatment groups. After each mouse was anesthetized with isoflurane inhalation, the mouse was fixed in a lateral position for eyeball blood collection. Following this, cervical dislocation was performed for euthanasia, and the mouse was then immediately dissected. The intestine and gastrocnemius (GAS) tissues and plasma were obtained to perform the following experiments (Figure 1).

### 2.2. Measure of Blood Glucose

As is widely known, stress can cause increased blood glucose. To verify whether a high temperature induced stress, blood was obtained from the tail of the mice (we used a mouse tail vein injection device (ZH-XWZY, Anhui Zhenghua Biologic Apparatus Facilities, Huaibei, China) to fix the mouse, and collect 50 µL of blood from the mouse tail with a blood collection needle), and the samples were measured using a blood glucose meter (GC14906201, Roche, Basel, Switzerland) according to the manufacturer’s protocol, before the mice were sacrificed.

### 2.3. Measurements of Antioxidant Activity

GAS tissues were homogenized in a 0.9% saline solution using a homogenizer. To obtain the supernatant, the samples were centrifuged for 20 min at a rotational speed of 2000 g. The total protein concentration of the supernatant was determined using a bicinchoninic acid (BCA) protein assay kit (P0011, Beyotime, Shanghai, China). To assess the antioxidant capacity, the activities of the total superoxide dismutase (T-SOD) (S0101, Beyotime), glutathione peroxidase (GSH-Px) (S0056, Beyotime), and MDA (S0131, Beyotime) levels were quantified based on WST-8, NADPH, and TBA using colorimetric methods. The values of T-SOD (wavelengths: 550 nm) and GSH-Px (wavelengths: 412 nm) were expressed as the units per milligram of protein. The values of MDA (wavelengths: 532 nm) were expressed as the micromoles per milligram of protein. At least three biological replicates were used.

### 2.4. Hematoxylin and Eosin (H&E) Staining

First, 4% paraformaldehyde (PFA) was used to fix GAS tissues. After 48 h, the tissues were dehydrated and then embedded in paraffin wax. The tissues were sliced into sections 5 μm thick. After dewaxing, the tissue sections were stained with H&E and sealed with neutral gum. Images of the sections were obtained using a microscope (BX51, Olympus, Tokyo, Japan), and the villus length (V), crypt depth (C), and average cross-sectional area (CSA) of the muscle fibers were measured using Image J software (Version 1.4, Bethesda, Rockville, MD, USA). Additionally, the villus length/crypt depth (V/C) ratio was calculated. At least three biological replicates (3 mice) were used.

### 2.5. Immunofluorescence Staining

GAS tissues were fixed with 4% PFA and embedded in paraffin wax, which was then cut into tissue sections. Then, the tissue sections were dewaxed and pretreated with goat serum (SL038, Solarbio, Beijing, China). Subsequently, they were incubated with mouse antibodies against a myosin heavy chain 1 (MYH1) monoclonal antibody (67299-1-Ig, 1:200, Proteintech, Wuhan, China), rabbit antibodies against a myosin heavy chain 7 (MYH7)-specific polyclonal antibody (22280-1-AP, 1:200, Proteintech), or rabbit antibodies against a lysosome-associated membrane protein 1 (LAMP1) monoclonal antibody (99437S, 1:200, Cell Signaling Technology, Danvers, MA, USA) overnight at 4 °C. The next day, these sections were left at room temperature for 30 min to equilibrate to ambient temperature. After washing in phosphate-buffered saline (PBS), they were incubated in goat anti-mouse IgG H&L (Alexa Fluor ^®^594, ab150116, 1:500, Abcam, Cambridge, UK) or goat anti-rabbit IgG H&L (Alexa Fluor ^®^488, ab150077, 1:500, Abcam) for 2 h at room temperature. After washing in PBS, the nuclei were labeled using 4′,6-Diamidino-2′-phenylindole (DAPI). Images of the sections were obtained using a fluorescence microscope, and the number of positive cells was measured using Image J software. At least three biological replicates were used.

### 2.6. Enzyme-Linked Immunosorbent Assay (ELISA)

Plasma was collected from the eye of the mice to measure the corticosterone (CORT) and tumor necrosis factor (TNF-α) concentrations using two competitive ELISA kits: the mouse CORT ELISA kit (SL07863, SLCY, Beijing, China) and the mouse TNF-α ELISA kit (SL08798, SLCY). The procedure for the detection of CORT and TNF-α in the plasma was performed according to the manufacturer’s instructions. These OD values were detected using an enzymoscope, and the contents of CORT and TNF-α in the plasma were calculated according to the standard curve. The inter-experiment CV was <12%, and the intra-experiment coefficient of variation (CV) was <10%. At least three biological replicates were used.

### 2.7. Quantitative Real-Time Polymerase Chain Reaction (qRT-PCR)

The total RNA of GAS tissues was extracted with a Trizol reagent (9109, Takara, San Jose, CA, USA). A HiScript III QRT supermix (R323-01, Vazyme, Nanjing, China) was used to synthesize the cDNA, which acted as a template for the qRT-PCR experiment. qRT-PCR was performed with an SYBR green master mix (MQ10201, Monad, Suzhou, China). Fluorescence signals were obtained, and the cycle threshold (Ct) was measured on a OneStep Plus instrument (Applied Biosystems, Carlsbad, CA, USA). Gene expression was shown by calculating the average Ct ratio relative to the housekeeping gene β-actin. At least three biological replicates were used. The primers used are shown in Table 1.

### 2.8. Western Blotting

The proteins of GAS tissue and C2C12 cells were obtained using an RIPA lysis buffer (P0013B, Beyotime) containing 1% protease inhibitor cocktail (G2008, Servicebio, Wuhan, China). The total protein concentrations of the GAS tissues and C2C12 cells were measured using a BCA protein assay kit (P0011, Beyotime). The proteins were separated from the tissues and cells using 10% sodium dodecylsulfate–polyacrylamide gel electrophoresis (SDS-PAGE). Then, the protein bands were electro-blotted onto polyvinylidene fluoride membranes (PVDF, ISEQ00010, Millipore, Burlington, MA, USA). The PVDF membranes were blocked in 2.5% fat-free dry milk (prepared with TBST) for 90 min. Next, they were incubated in rabbit antibodies against a myogenic differentiation 1 (MyoD1) antibody (ab133627, 1:1000, Abcam), an SQSTM1/p62 monoclonal antibody (A19700, 1:1000, ABclonal), an LC3B monoclonal antibody (ab192890, 1:1000, Abcam), and a GAPDH polyclonal antibody (10494-1-AP, 1:20,000, Proteintech), and rabbit antibodies against a lysosome-associated membrane protein 1 (LAMP1) monoclonal antibody (1:1000, 99437S, Cell Signaling Technology) or mouse antibodies against a myogenin (MyoG) monoclonal antibody (SC-12732, 1:1000, Santa Cruz biotechnology, Dallas, TX, USA) and paired box 7 (Pax7) monoclonal antibody (ab199010, 1:1000, Abcam) overnight at 4 °C. These membranes were washed in TBST and then incubated in horseradish peroxidase (HRP)-conjugated goat anti-rabbit IgG (AS014, 1:25,000, ABclonal, Woburn, MA, USA) or HRP-conjugated goat anti-mouse IgG (SA00001-1, 1:25,000, Proteintech) for 2 h at 37 °C. The intensities of the protein bands were quantified using Image J. The protein levels were normalized to the density ratio of GAPDH. In this study, the relative protein level in the control group was defined as 100%. At least three biological replicates were used.

### 2.9. Measurement and Analysis of Gut Microbiota

Fresh colon contents were obtained from the mice and mixed with sterile PBS. After centrifugation, we extracted the microbial DNA using the Hipure Soil DNA Kits (Magen, Guangzhou, China), and the procedures were performed according to the manufacturer’s instructions. PCR was used to amplify the 16S rDNA target region (V3-V4 region, universal primers: 341F, CCTACGGGNGGCWGCAG; 806R, GGACTACHVGGGTATCTAAT) of the ribosomal RNA genes. After amplification, 2% agarose gels were used to observe the amplicons, which were then purified using AMPure XP Beads (Beckman, CA, USA) according to the manufacturer’s instructions. Next, these purified products were used to build sequence libraries. The Illumina DNA Prep Kit (Illumina, CA, USA) was used to establish sequencing libraries according to the manufacturer’s protocols. Additionally, the quality of the library was assessed using the ABI StepOnePlus Real-Time PCR System (Life Technologies, Foster City, CA, USA). After confirming the availability of the sequence, sequencing was performed on the Novaseq 6000 platform, producing 2 × 250 bp paired-end reads. However, the raw reads were low quality and had to be filtered to obtain high-quality clean reads, which was performed using FASTP (version 0.18.0) [26]. Operational taxonomic units (OTU) with ≥97% similarity were obtained by clustering the final tags using the UPARSE pipeline (Version 9.2.64) [27]. In this process, the chimeric tags can affect the result; therefore, all chimeric tags were deleted using the UCHIME algorithm [28] to produce effective tags. Meanwhile, the effective tags were obtained for further analysis. Using the R project VennDiagram package (version 1.6.16), a between-group Venn analysis showed the differences in the OTUs among the control, HS 3 d, HS 7 d, and HS 14 d groups. Species comparison among the groups was conducted using multiple methods, including Welch’s *t*-test and the Wilcoxon rank test, which are included in the R project Vegan package (version 2.5.3). Tukey’s HSD test and the Kruskal–Wallis H test were also used in the same package to analyze the species comparison among the groups. Alpha index comparison among the groups was also performed using the Wilcoxon rank test and Welch’s *t*-test in the R project Vegan package (same version as above). The tag sequence with the highest abundance was chosen as the typical sequence in each cluster. To observe differences in the types of species among the groups, QIIME (version 1.9.1) was used to calculate the Chao1, ACE, and Shannon indices [29]. Non-metric multi-dimensional scaling (NMDS) and principal coordinates analysis (PCoA) of (un)weighted UniFrac, Jaccard, and Bray–Curtis distances were also generated using the R project Vegan package (same version as above) and drawn using the R project ggplot2 package (version 2.2.1). DNA library sequencing was performed on the Illumina NovaSeq 6000/PacBio Revivo platform (Gene Denovo Biotechnology Co., Ltd., Guangzhou, China). Our sequencing data were uploaded onto the Sequence Read Archive of the National Center for Biotechnology Information (NCBI) (Bioproject: PRJNA1156015) for publication.

### 2.10. Cell Culture and Treatment

In order to explore whether oxidative stress induced by heat stress regulates skeletal muscle development through autophagy, we conducted further verification through cell culture experiments. C2C12 cells were cultured in Dulbecco’s modified Eagle’s medium (DMEM) (SH30022.01, Cytiva, Marlborough, MA, USA) supplemented with 10% fetal bovine serum (FBS) (U11-020A, YOBIBIO, Shanghai, China) and 100 U/mL penicillin and streptomycin (P1400, Solarbio Life Sciences, Beijing, China), and maintained at 37 °C in a 5% CO_2_ humidified incubator. The C2C12 cells were seeded on 12-well plates (5 × 10^5^ cells/mL), which were treated with 200 μM H_2_O_2_ (H_2_O_2_ group). Additionally, 5 mM NaB (303,410, Sigma-Aldrich, St. Louis, MO, USA) was used to treat the C2C12 cells treated with 200 μM H_2_O_2_ (H_2_O_2_ + NaB). Some cells were sequentially treated with inhibitors of autophagy initiation (0.2 μM wortmannin, MedChemExpress LLC, H_2_O_2_ + NaB + Wort-cells), NaB, and H_2_O_2_. Finally, the C2C12 cells were collected for Western blotting analysis. Each assay was repeated three times (using independent cell culture experiments).

### 2.11. Statistical Analysis

All data were presented as the mean ± SEM, and analyzed using SPSS 27 (SPSS Inc., Chicago, IL, USA). Graphics were produced using Graphpad Prism 9 software (La Jolla, San Diego, CA, USA). During the data acquisition process, the intestinal morphological test requires that the sampled intestinal tissue is free of contents, and the same location of the tissue is used for different experiments. Additionally, under conditions such as the loss of ear tags and the limitations of blood sample volume collection, the experiments were performed using at least three independent biological repeats (*n* ≥ 3 mice or *n* ≥ three times for independent cell experiments). The analysis method used for 16S sequencing was the same as that described in Section 2.9. Two-tailed Student’s *t*-tests were used to compare the data with a corresponding group (groups = 2). For comparisons against the HS group, #, *p* < 0.05, and ###, *p* < 0.001. Variations among the groups (groups > 2) were assessed using one-way analysis of variance (ANOVA) followed by Dunnett’s *t*-test (two-tailed) compared to the control group, with statistical significance indicated as follows: *, *p* < 0.05; **, *p* < 0.01; ***, *p* < 0.001; and ****, *p* < 0.0001, or displaying the *p*-value on the data chart of HS or H_2_O_2_. For comparisons against the HS group or H_2_O_2_ group, #, *p* < 0.05; ##, *p* < 0.01; and ###, *p* < 0.001, or displaying the *p*-value on the data chart of HS + NaB or H_2_O_2_ + NaB. For comparisons against the H_2_O_2_ + NaB group, &, *p* < 0.05.

### 2.12. Blinding

Blinding was implemented during the outcome assessment and data analysis stages. The investigator responsible for data collection and analysis was unaware of the group allocation to prevent potential bias.

## 3. Results

### 3.1. Heat Stress Affected Morphological Characteristics of Intestine and Skeletal Muscle

**Figure 2:**
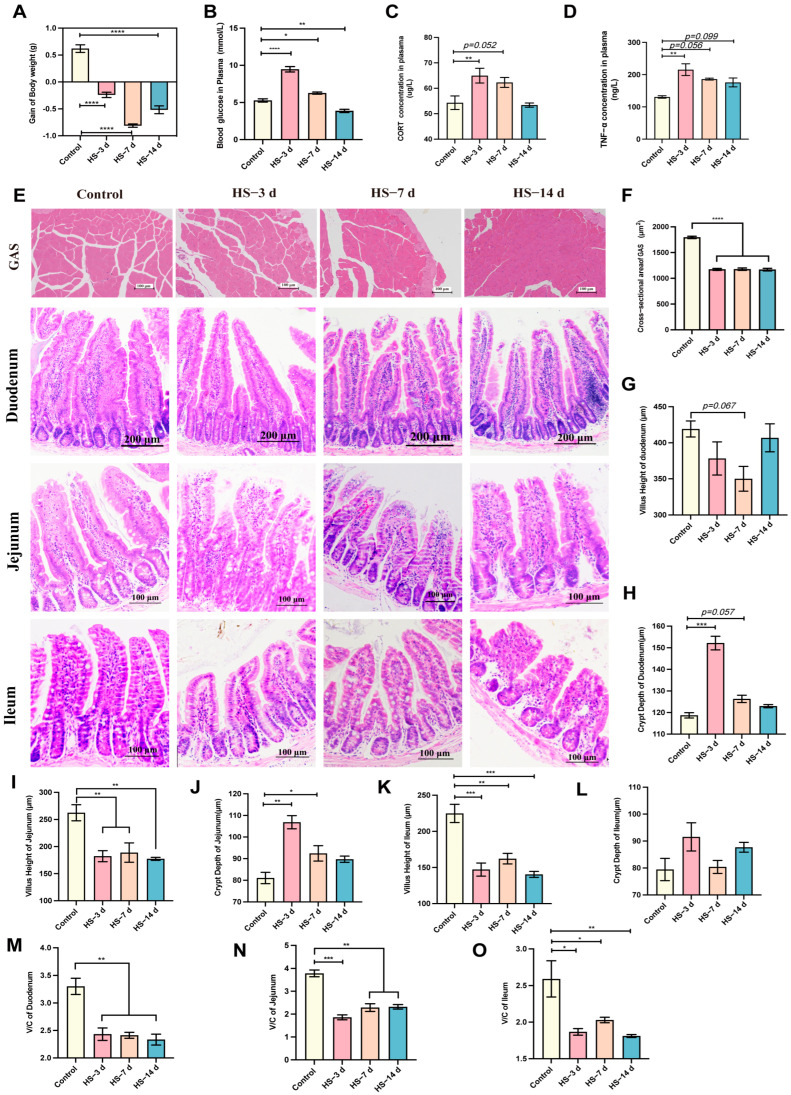
Heat stress (HS) affected the morphological characteristics of the intestine and skeletal muscle. (**A**) Body weight gain (*n* = 3). (**B**) Blood glucose levels (*n* = 4). (**C**) The secretion of the stress hormone CORT (*n* = 6). (**D**) The concentration of TNF-α in plasma (*n* = 3). (**E**) Gastrocnemius (GAS, scale bar =100 μm), duodenum (scale bar = 200 μm), jejunum (scale bar =100 μm), and ileum (scale bar =100 μm) histopathology (H&E staining) after 3 d, 7 d, and 14 d of HS (*n* = 3). (**F**) The statistical analysis of the average cross-section of GAS. (**G**,**H**) The analysis of villus (**G**) and crypt depth (**H**) of the duodenum (*n* = 3). (**I**,**J**) Analysis of the villus (**I**) and crypt depth (**J**) of the jejunum (*n* = 3). (**K**,**L**) Analysis of the villus (**K**) and crypt depth (**L**) of the ileum (*n* = 3). (**M**–**O**) Analysis of the V/C ratio of the duodenum (**M**), jejunum (**N**), and ileum (**O**) (*n* = 3). The data are shown as the means and standard errors (mean ± SEM). *, *p* < 0.05; **, *p* < 0.01; ***, *p* < 0.001; and ****, *p* < 0.0001 compared with the control group or displaying the *p*-value on the data chart of the HS group. HS, heat stress; V/C, villus height/crypt depth; CORT, corticosterone; TNF-α, tumor necrosis factor-alpha.

To better explore the response of the mice to high temperature, different high-temperature exposure times were implemented in the experiments. The results show that the high temperature significantly reduced the body weight gain of the mice after 3 d, 7 d, and 14 d (Figure 2A). The blood glucose levels were obviously increased after 3 d and 7 d but not after 14 d, indicating adaptation over time and even hypoglycemia (Figure 2B). Additionally, the concentration of CORT was increased under high temperature after 3 d and 7 d (Figure 2C). Inflammation was induced, and the levels of TNF-α were increased after 3 d, 7 d, and 14 d (Figure 2D). Thus, the high temperature induced a stress response and inflammation in the mice.

HS may have influenced the homeostasis of the mice’s intestines and skeletal muscles, as shown in their morphological characteristics. In our study, the cross-sectional area of the GAS muscle was used as an indicator of skeletal muscle function. The results show significant decreases in the average cross-sectional area after 3 d, 7 d, and 14 d of HS (Figure 2E,F), which affected the skeletal muscle function. Additionally, the villus height, crypt depth, and V/C ratio of the intestine reflect its ability to digest and absorb. The results show that the villus height of the duodenum was decreased after 7 d under HS, and the crypt depth of the duodenum was increased after 3 d and 7 d under HS (Figure 2E,G,H). The villus height of the jejunum was significantly decreased after 3 d, 7 d, and 14 d under HS, and the crypt depth of the jejunum was increased after 3 d and 7 d under HS (Figure 2E,I,J). The villus height of the ileum was significantly decreased after 3 d, 7 d, and 14 d under HS, but the crypt depth of the ileum was not significantly changed (Figure 2E,K,L). Additionally, the V/C ratio of the duodenum, jejunum, and ileum were significantly decreased after 3 d, 7 d, and 14 d under HS (Figure 2M–O). Therefore, HS affected the morphology of the intestine and skeletal muscle, which may have influenced their function.

### 3.2. Heat Stress Affected the Gut Microbiota Composition of Mice

**Figure 3:**
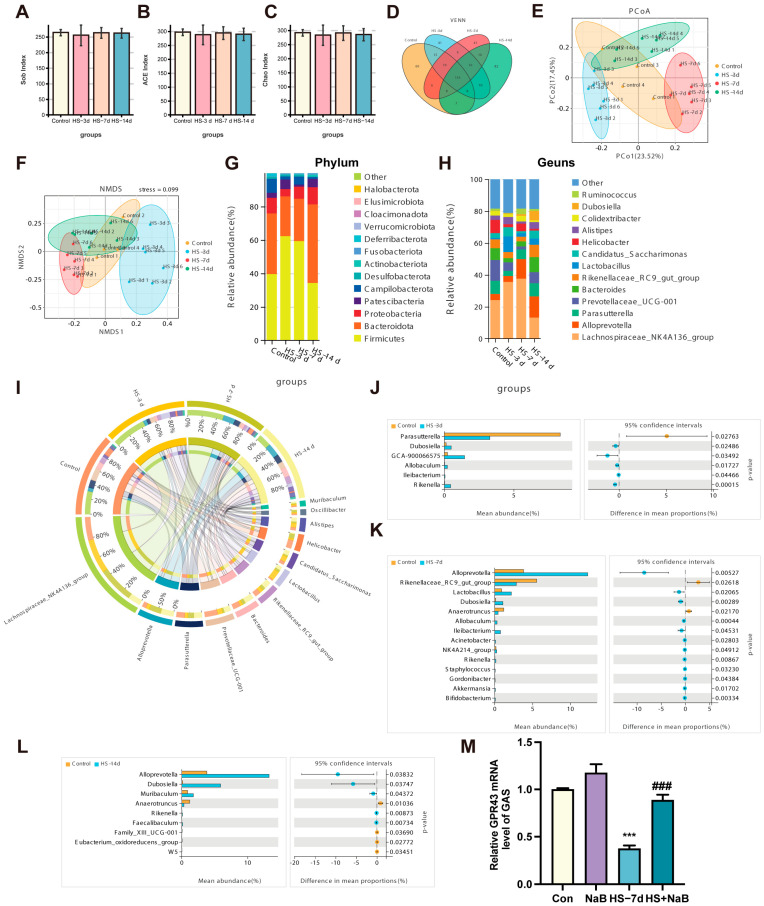
Changes in the colonic microbial composition of the mice stimulated by heat stress. (**A**–**C**) The Sob index (**A**), ACE index (**B**), and Chao index (**C**) of control, HS 3 d, HS 7 d, and HS 14 d groups (*n* = 4–6). The β-diversity shows the distribution of each sample in the four groups (*n* = 4–6). (**D**) A Venn diagram comparing and analyzing the relationship between different OTU in each group (*n* = 4–6). (**E**,**F**) A PCoA score plot and NMDS score plot based on the Bray–Curtis score plot, which were calculated based on the OTUs in the colon (*n* = 4–6). (**G**) Species distribution in columnar format, showing the relative contribution of the top 13 phyla in the groups in the colon (*n* = 4–6). (**H**) Relative abundance of the top 13 genera in these groups in the colon (*n* = 4–6). (**I**) A Circos plot showing the relationships between the microbiota and the groups (*n* = 4–6). (**J**–**L**) The species differences between the control group and the HS group (HS 3 d, HS 7 d, and HS 14 d) were analyzed using Welch’s *t*-test, *p* < 0.05 (*n* = 4–6). (**M**) The level of GPR43 mRNA (*n* = 3). ***, *p* < 0.001 compared with the control group. ###, *p* < 0.001 compared with the HS group.

To verify the influence of HS on gut microflora, the effects of different durations of HS on the colonic microflora of mice were explored using 16S rRNA gene amplicon sequencing. The results show that no significant changes occurred in the Chao1, ACE, and Shannon indices among the four groups (Figure 3A–C). The Venn diagram illustrates that different durations of HS treatments resulted in different changes in the operational taxonomic units (OTUs). There were 70 kinds of OTUs specific to the HS 3 d group, 70 kinds of OTUs specific to the HS 7 d group, and 72 kinds of OTUs specific to the HS 14 d group (Figure 3D) compared with the control group. In the four groups of microbial populations, analysis of the β-diversity and clustering patterns on PCoA and NMDS plots showed an obvious clustering of microbiota composition (Figure 3E,F). Specifically, we characterized the influence of HS on the relative abundance of different bacterial taxa at the phylum and genus levels (Figure 3G,H). The distinct differences in the phylum and genus distributions were observed on the relative abundance map. Meanwhile, we used Circos maps to show the proportion of dominant species in different groups and their distribution among different groups (Figure 3I). The result of Welch’s *t*-test shows that the parasutterella was obviously reduced, while Dubosiella, Allobaculum, Ileibacterium, and Rikenella were significantly increased in the HS 3 d group compared with the control group (Figure 3J). Allpprevotella, Lactobacillus, Dubosiella, Allobaculum, IIeibacterium, Acinetobacter, Rikenella, Staphylococcus, Gordonibacter, Akkermansia, and Bifidobacterium were obviously increased in the HS 7 d group compared with the control group. The Rikenellaceae RC9 gut group and Anearotruncus were significantly reduced in the HS 7 d group compared with the control group (Figure 3K). Alloprevotella, Dubosiella, Muribaculum, Rikenella, and Feacalibaculum were significantly increased in the HS 14 d group compared with the control group. Anaerotruncus and the Eubacterium oxidoreducens group were significantly reduced in the HS 14 d group compared with the control group (Figure 3L). All together, these results indicate that the HS severely aggravated the effect on the taxonomic composition of the gut microbiota. HS caused changes in the gut microbes, and the number of microorganisms with significant differences was the highest after 7 days. As mentioned above, the intestinal flora that produces SCFAs was disrupted, which may have led to reduction in their production. As a beneficial metabolite of microorganisms, SCFAs participate in the development of the body. The results of our study show that HS can reduce the level of G Protein-Coupled Receptor 43 (GPR43) mRNA, and the addition of NaB can increase the level of GPR43 mRNA in skeletal muscle (Figure 3M). Therefore, butyrate may regulate skeletal muscle homeostasis.

### 3.3. NaB Reduced the Oxidative Stress Induced by HS and Stabilized the Development of Skeletal Muscle

**Figure 4:**
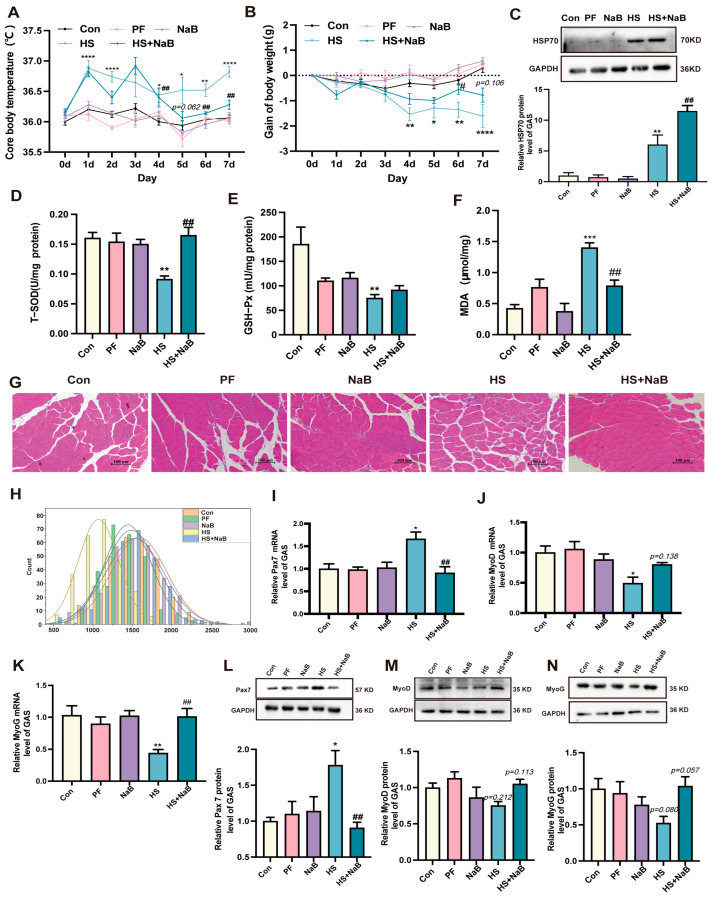
NaB reduced the oxidative stress induced by HS and stabilized the development of skeletal muscle. (**A**) The core body temperature (*n* = 4–5). (**B**) Changes in body weight (*n* = 5). (**C**) Representative Western blotting images and statistical analysis of HSP70 protein (*n* = 3). (**D**–**F**) The concentration of T-SOD (**D**), GSH-Px (**E**), and MDA (**F**) of skeletal muscle in mice (*n* = 3). (**G**,**H**) Representative images of GAS H&E staining (**G**) and statistical analysis of the cross-section of GAS fibers (**H**) (*n* = 3), scale bar = 100 μm. (**I**–**K**) The relative levels of Pax7 (**I**), MyoD (**G**), and MyoG (**K**) mRNA (*n* = 3–5). (**L**–**N**) Representative images and statistical analysis of Pax7 (**L**), MyoD (**M**), and MyoG (**N**) protein (*n* = 3). *, *p* < 0.05; **, *p* < 0.01; ***, *p* < 0.001; and ****, *p* < 0.0001 compared with the control group or displaying the *p*-value on the data chart of the HS group. #, *p* < 0.05 and ##, *p* < 0.01 compared with the HS group or displaying the *p*-value on the data chart of the HS + NaB group.

As described above, the effects of NaB on skeletal muscle development in HS mice were investigated in this study. Our results indicate that the NaB significantly decreased the elevated temperature caused by HS in the mice (Figure 4A), which demonstrates that HS can regulate body temperature. Additionally, NaB protected the mice from HS-induced weight loss, helping maintain body weight homeostasis (Figure 4B). After HS, the HSP70 protein level was significantly increased. NaB further increased the level of HSP70, helping to better regulate the response to HS (Figure 4C). Therefore, NaB can resist the negative influence that HS has on body temperature and weight.

**Figure 5:**
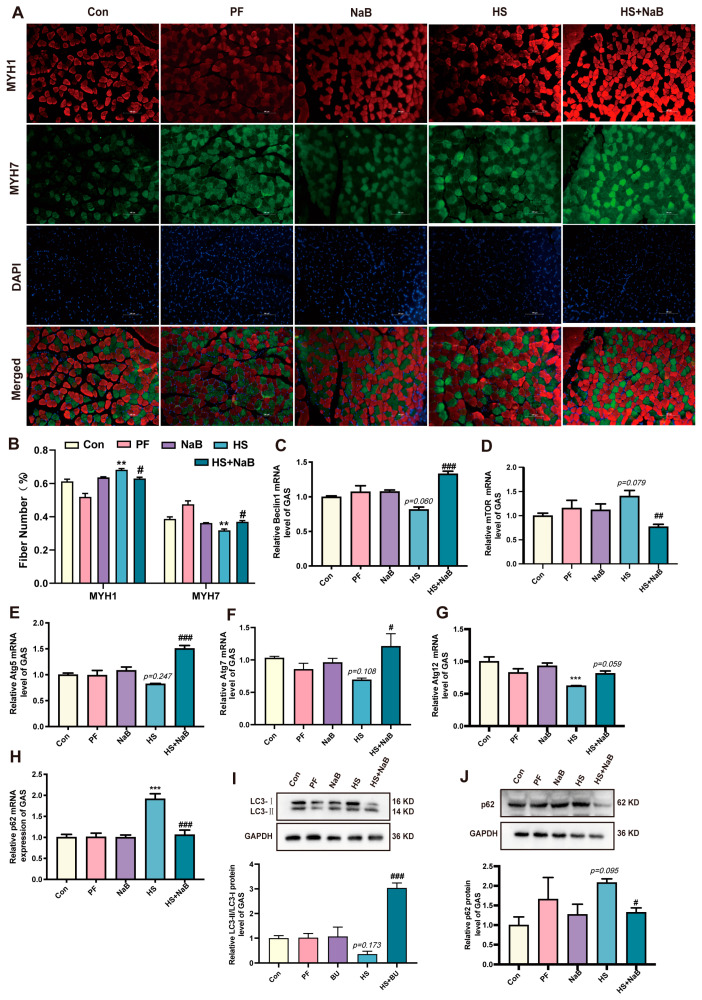
NaB prevents muscle fiber type conversion and autophagy inhibition induced by heat stress. (**A**) Representative images of MYH1 and MYH7 protein immunofluorescence staining. (**B**) Analysis of the positive cell number of MYH1 and MYH7 protein. (**C**–**H**) The relative levels of Beclin1 (**C**), mTOR (**D**), Atg5 (**E**), Atg 7 (**F**), Atg 12 (**G**), and p62 mRNA (**H**) (*n* = 3). (**I**,**J**) Representative images and analysis statistical chart of LC3II/LC3I (**I**) and p62 (**J**) protein (*n* = 3). **, *p* < 0.01 and ***, *p* < 0.001 compared with the control group or displaying the *p*-value on the data chart of the HS group. #, *p* < 0.05; ##, *p* < 0.01; and ###, *p* < 0.001 compared with the HS group or displaying the *p*-value in the HS + NaB group.

In addition, stress can disrupt the balance between oxidative and antioxidative processes. In our study, the levels of total superoxide dismutase (T-SOD) and GSH-Px were significantly decreased under HS, but, the level of MDA was obviously increased. However, NaB significantly increased the levels of antioxidants (T-SOD) and decreased the level of MDA after HS (Figure 4D–F). The cross-sectional area of GAS was decreased after HS; however, NaB increased it under HS (Figure 4G,H), which indicates that NaB can help maintain the homeostasis of skeletal muscle. The influence of NaB on regeneration and proliferation in skeletal muscle under HS remains unclear. The study results show that the Pax7 mRNA and protein levels were significantly increased after HS, suggesting that it activated the repair function of skeletal muscle. However, after NaB administration, the Pax7 mRNA expression level recovered to the level of the control group (Figure 4I,L). Additionally, the mRNA and protein levels of MyoD and MyoG were decreased under HS. However, NaB was able to reverse these effects (Figure 4J,K,M,N). MYH1 and MYH7 were labeled in the GAS cells using immunofluorescence staining, and the results suggest that the number of fibers labeled with MYH1 was significantly increased; however, NaB reduced this number (Figure 5A,B). In contrast, HS obviously decreased the number of fibers labeled with MYH7, but NaB reversed this effect (Figure 5A,B). These findings suggest that HS weakened the function of skeletal muscle by facilitating the transition of slow fibers to fast fibers; however, NaB protected the skeletal muscle from these effects.

### 3.4. NaB Reversed the Inhibition of Skeletal Muscle Autophagy Caused by Heat Stress

**Figure 6:**
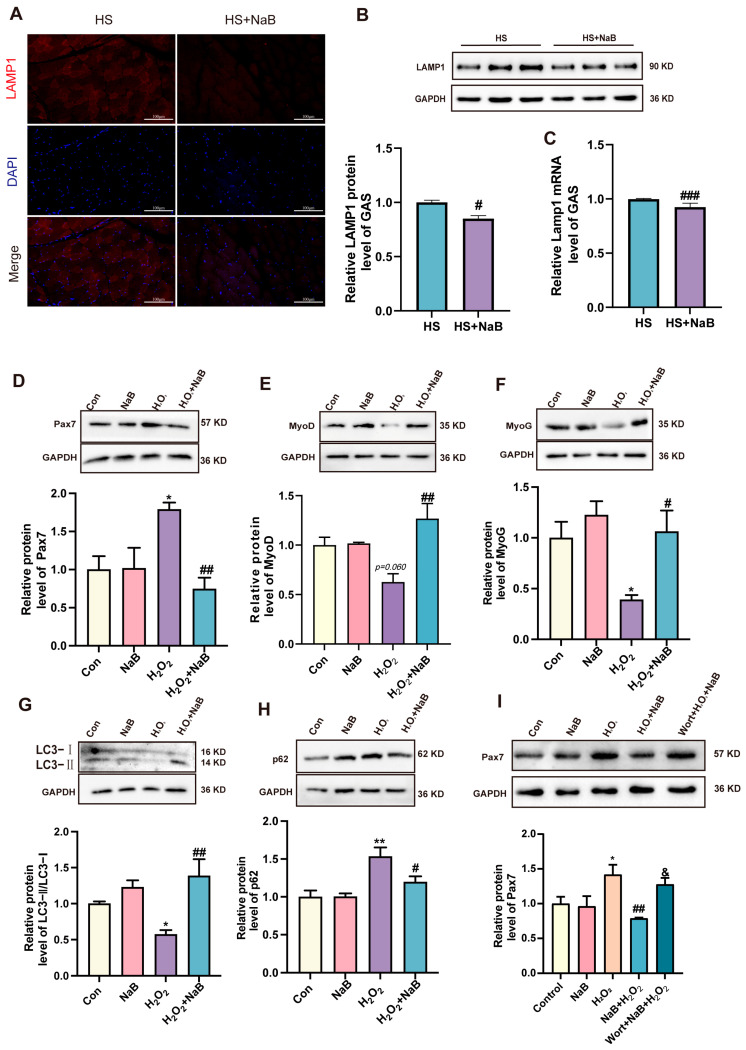
NaB alleviated C2C12 dysplasia induced by oxidative stress by up-regulating autophagy. (**A**) Representative images of LAMP1 protein immunofluorescence staining in GAS. (**B**) Representative images and statistical analysis of LAMP1 protein in GAS (*n* = 3). (**C**) The relative level of lamp1 mRNA in GAS (*n* = 3). (**D**) Representative images and statistical analysis of Pax7 protein (*n* = 3). (**E**) Representative images and statistical analysis of MyoD protein (*n* = 3). (**F**) Representative images and statistical analysis of MyoG protein (*n* = 3). (**G**) Representative images and statistical analysis of LC3II/LC3I protein (*n* = 3). (**H**) Representative images and statistical analysis of p62 protein (*n* = 3). (**I**) Representative images and statistical analysis of Pax7 protein (*n* = 3). *, *p* < 0.05 and **, *p* < 0.01 compared with the control group or displaying the *p*-value on the data chart of the H_2_O_2_ group. #, *p* < 0.05; ##, *p* < 0.01; and ###, *p* < 0.001 compared with the H_2_O_2_ group. &, *p* < 0.05 compared with the NaB + H_2_O_2_.

HS can affect autophagy, but it is unclear whether NaB can reverse autophagy changes in skeletal muscle under HS. Therefore, in our study, the effects of NaB on the autophagy level under HS were investigated. The results show that the mRNA levels of Beclin1 were decreased under HS. However, NaB reversed this effect (Figure 5C). Additionally, the mTOR mRNA level was increased under HS, but the NaB reduced the mTOR mRNA level (Figure 5D). Furthermore, the mRNA levels of autophagy-related gene 5 (Atg5), Atg7, and Atg12 were decreased under HS; however, NaB increased these mRNA levels (Figure 5E–G). The mRNA level of p62 was significantly increased under HS. In contrast, NaB reversed its level (Figure 5H). The ratio of LC3II/LC3I was decreased after HS, but NaB significantly reversed this effect (Figure 5I). Additionally, the p62 protein level was increased under HS; however, NaB significantly decreased it (Figure 5J). NaB weakened the positive signaling of LAMP1 compared with the HS group (Figure 6A). Furthermore, NaB decreased the LAMP1 protein level (Figure 6B) and lamp1 mRNA level (Figure 6C) compared with the HS group. These results indicate that NaB can reverse the inhibition of autophagy initiation and the binding of autophagosomes to lysosomes in skeletal muscle under HS.

### 3.5. NaB Alleviated C2C12 Dysplasia Induced by Oxidative Stress by Up-Regulating Autophagy

NaB alleviated HS-induced oxidative stress and skeletal muscle dysplasia and reversed the HS-induced autophagy inhibition of skeletal muscle cells. However, whether NaB regulates skeletal muscle development through autophagy remains unclear. C2C12 cells were treated with H_2_O_2_ to mimic oxidative stress in our study. The result shows that the level of Pax7 protein was obviously increased due to treatment with H_2_O_2_. Then, NaB reversed its levels (Figure 6D). The MyoG protein level was obviously decreased, and the MyoD protein level was decreased when the C2C12 cells were treated with H_2_O_2_, but NaB significantly recovered these protein levels (Figure 6E,F). The ratio of LC3II/LC3I was decreased after oxidative stress and the level of p62 protein was significantly increased under oxidative stress. However, NaB reversed these effects (Figure 6G,H). Then, we inhibited autophagy by adding wortmannin to the NaB + H_2_O_2_ group. The results show that the level of Pax 7 protein was increased compared with the NaB + H_2_O_2_ treatment (Figure 6I). Therefore, the activation of autophagy is the mechanism by which NaB alleviates oxidative stress-induced skeletal muscle dysplasia.

## 4. Discussion

Heat stress (HS) is a widespread global environmental problem that causes physical exhaustion, including skeletal muscle weakness and the disruption of intestinal homeostasis [7,30,31]. The intestinal–skeletal muscle axis is an area of ongoing research. For example, butyrate was shown to improve muscle growth performance in piglets [19], and it can influence skeletal muscle metabolism [32]. NaB ameliorated diabetes-related sarcopenia through the ILC2s/IL-13/STAT3 pathway [33], and dietary NaB supplementation promoted the expressions of myosin heavy chain I (MyHCI) and MyHCIIα to promote oxidative fiber growth in skeletal muscle [34]. SCFAs play an integral role in skeletal muscle metabolism and function [35]. However, the mechanism by which intestinal microbial metabolites improve skeletal muscle development under HS conditions remains unclear. In this study, the body weight of mice decreased under HS, but NaB increased the body weight under HS. Additionally, NaB also reduced the increased core temperature of the mice under HS. The high temperature increased the levels of CORT and glucose in plasma, which demonstrates that stress was induced under the high temperature. Other research showed that HS can induce an inflammation response [36]. In our study, the concentration of TNF-α was increased under HS. Therefore, HS disrupts the body homeostasis.

As is widely known, the imbalance of body homeostasis can lead to metabolism disorders. Intestinal and skeletal muscles are the most important metabolic organs of the body. Related research suggested that HS can induce intestinal damage and destroy intestinal integrity [37]. In our study, the intestinal villus height was decreased and the crypt depth was increased. Furthermore, the composition of gut microbiota was disrupted under HS, which demonstrates that the function of the intestine may be disrupted by HS. The protective effect of dietary butyrate on intestinal damage induced by HS was previously shown [37]. However, the function of NaB in protecting skeletal muscle from HS was unclear. In this study, NaB, the most important metabolite of gut microbes, was added to explore the function of how it protects skeletal muscle from HS. Firstly, the receptor of butyrate was decreased under HS, but NaB increased the expression of GPR43, which demonstrates that NaB can adjust the homeostasis of skeletal muscle development. Evidence showed that, in response to HS, heat shock proteins play a vital role in regulating the rate and efficiency of muscle development [38]. The results of our study show that HS activated the expression of HSP70, which was higher in the HS group when NaB was administered. HSP regulated the physiological mechanism of HS adaptation [39]. Therefore, NaB can help animals and humans adapt to HS. Meanwhile, HS decreased the concentration of T-SOD and GSH-Px and increased the levels of MDA in skeletal muscle. NaB improved antioxidant levels under HS. In another study, HS affected metabolites in the longissimus dorsi muscle and led to changes in the endogenous antioxidant defense of finishing pigs [40]. Therefore, HS causes oxidative stress in skeletal muscle. The addition of butyrate to maternal rats enhances mitochondrial biogenesis in the skeletal muscles of their weaned offspring [41], helping to maintain a balance between oxidative and antioxidative processes. In addition, the developmental homeostasis of skeletal muscle also depends on muscle-related factors, including Pax7, MyoG, and MyoD. Another study showed that chronic HS disrupted protein metabolism in breast muscle, which affected the development of skeletal muscle [42]. Pax7 is a marker of skeletal muscle regeneration, which was activated under HS in our study. However, NaB reduced the protein level of Pax7 to regain normal homeostasis of the skeletal muscle. Also, HS decreased the protein level of MyoD and MyoG, which disrupted the development of skeletal muscle. However, NaB increased the expression of MyoG and MyoD. Some studies have also shown that HS can change the muscle protein composition, the muscle’s response to HS showing specificity for fiber type [43]. Continuous mild HS was found to induce changes in fiber type from fast to slow [44], and increased butyrate can increase the levels of slow-twitch fibers [45]. In our study, the results of immunofluorescence staining show that NaB increased the number of slow muscle fibers (MYH7) and reduced the number of fast muscle fibers (MYH1) under HS, which is consistent with the results of previous research [44,45]. Therefore, under HS conditions, NaB can better maintain the developmental homeostasis of skeletal muscle.

However, the signaling pathway through which butyrate regulates skeletal muscle development is unclear. A study showed that high temperature prevented the initiation of autophagy and the degradation of autophagosomes [46]. In this study, HS decreased the gene expression of autophagy-related factors, including Beclin1, Atg7, Atg5, and Atg12, and increased the p62, lamp1, and mTOR mRNA levels, which suggests that autophagy was inhibited. However, autophagy was activated and autophagic flow was normalized by adding NaB under HS. Therefore, HS inhibited autophagy, causing the abnormal development of skeletal muscle, but this effect was reversed by NaB. In vitro, C2C12 cells were treated with H_2_O_2_ to mimic oxidative stress, while other C2C12 cells were treated with NaB under oxidative stress. The results show that NaB alleviated the effect that oxidative stress caused and promoted the initiation of autophagy. In this study, an inhibitor of autophagy was added to the C2C12 cells at the same time as the H_2_O_2_ and NaB treatment. The results show that NaB can regulate the homeostasis of C2C12 cells through autophagy under oxidative stress. Therefore, autophagy plays an important role in the development of skeletal muscle under HS alleviated by NaB.

## 5. Conclusions

Taken together, our data elucidate the specific pathway by which NaB protects skeletal muscle development from HS. In this study, HS affected the mice’s body weight, body temperature, stress response, and inflammation; their intestinal digestion and absorption levels were inhibited; and their intestinal microbial composition was disrupted. At the same time, HS promoted oxidative stress in the skeletal muscle and activated the skeletal muscle regeneration process, but it slowed down the skeletal muscle proliferation ability; NaB reversed these effects. These results indicate that HS inhibits the initiation of autophagy and the degradation of autophagosomes in skeletal muscle, and NaB can maintain the homeostasis of skeletal muscle development by activating autophagy.

## Figures and Tables

**Table 1:** Primer sequences.

Name	Forward (5′-3′)	Reverse (5′-3′)
*Pax7*	CCTGGGCGACAAAGGGAA	AGCTGCTCGGCTGTGAACG
*MyoG*	AACTACCTTCCTGTCCACCTTC	CACAGACTTCCTCTTACACACCT
*MyoD*	GAATGGCTACGACACCGCCTACTAC	ACGGGGTCTGGGTTCCCTGTT
*Atg5*	TGTGCTTCGAGATGTGTGGTT	ACCAACGTCAAATAGCTGACTC
*Atg7*	TGACCTTCGCGGACCTAAAGA	CCCGGATTAGAGGGATGCTC
*Atg12*	TAAACTGGTGGCCTCGGAAC	CCATCACTGCCAAAACACTCA
*Beclin1*	ATGGAGGGGTCTAAGGCGTC	TGGGCTGTGGTAAGTAATGGA
*mTOR*	GGCACACATTTGAAGAAGCAG	CTCGTTGAGGATCAGCAAGG
*GPR43*	ATCCTCCTGCTTAATCTGACCC	CGCACACGATCTTTGGTAGGT
*p62*	AGGATGGGGACTTGGTTGC	TCACAGATCACATTGGGGTGC
*Lamp1*	TGCTCCGGGATGCCACTAT	TGTTGTCCTTTTTCAGGTAGGTG
*β-actin*	TTGCTGACAGGATGCAGAAG	ACATCTGCTGGAAGGTGGAC

## Data Availability

The authors confirm that the data supporting the findings of this study are confidential within the article. In addition, 16S sequencing data can be obtained in the NCBI database, and the project number is PRJNA1156015. These data may be obtained from the corresponding author upon reasonable request.
